# Fibromyalgia: the Italian survey by the ministry of health

**DOI:** 10.1007/s10072-026-08882-z

**Published:** 2026-03-07

**Authors:** Alice Giordano, Davide Sasssos, Roberto Fancellu, Massimo Del Sette, Luca Boni, Alberto Sulli

**Affiliations:** 1 Clinical Epidemiology Unit, IRCCS Ospedale Policlinico San Martino, Genova, Italy; 2 Neurology Unit, IRCCS Ospedale Policlinico San Martino, Genova, Italy; 3 Clinical Epidemiology Unit, IRCCS Ospedale Policlinico San Martino, Genova, Italy; 4 Rheumatology Clinic Unit, IRCCS Ospedale Policlinico San Martino, Genova, Italy

**Keywords:** Fibromyalgia, Clinical pathways, National survey, Integrated care, Chronic pain

## Abstract

**Background:**

Fibromyalgia (FM) is a chronic condition with complex etiology, characterized by widespread pain and associated symptoms that significantly impact quality of life. In Italy, organizational heterogeneity and the absence of standardized care pathways hinder uniform management. The RF-2019-12369647 project was launched to develop a national strategy for integrated and multidisciplinary care.

**Methods:**

A nationwide mapping of clinical centers dedicated to FM was conducted through online reconnaissance and validation by Regional Health Departments. A structured survey was administered to 131 identified centers to collect data on organizational models, clinical workload, and care practices. In addition, a systematic literature review of diagnostic and therapeutic guidelines was performed, and a digital repository was developed to support information sharing.

**Results:**

A total of 131 centers were mapped, and 56 completed the survey (42.7% response rate). Centers managed an average of 485 patients annually, supported by multidisciplinary teams dominated by rheumatologists (91%). Despite territorial coverage, only 16% of centers were part of regional networks, and 21% reported not using any formal care protocol. Critical issues included lack of standardized pathways, insufficient training, and limited multidisciplinary integration. A cloud-based repository and web portal were developed to facilitate knowledge dissemination.

**Conclusions:**

The findings highlight wide regional disparities, fragmented care models, and insufficient adoption of guidelines for FM management in Italy. These results support the urgent need for a coordinated national strategy to ensure equitable, standardized, and multidisciplinary care pathways for patients with fibromyalgia.

## Introduction

Fibromyalgia (FM) is a chronic pain syndrome with a multifactorial pathophysiology, characterized by widespread musculoskeletal pain commonly accompanied by symptoms such as fatigue, sleep disturbances, cognitive complaints (“fibro fog”), autonomic dysfunction, and psychiatric comorbidities [1, 2], (e.g., mood and anxiety disorders). This heterogeneous clinical profile has a substantial impact on patients’ quality of life, psychosocial functioning, and work participation, contributing to the high complexity of care required for FM.

From a nosographic perspective, fibromyalgia was formally recognized by the World Health Organization (WHO) in 1992 and is included in the International Classification of Diseases, 10th Revision (ICD-10), under the code M79.7. Current evidence supports a multifactorial pathophysiology involving altered central pain processing, with additional contributions from peripheral nervous system dysfunction, including small fiber pathology, in a subset of patients.

In Italy, fibromyalgia is not yet included among the Essential Levels of Care (LEA) [3], although several regions (e.g., Tuscany, Veneto, Piedmont) have independently adopted dedicated diagnostic–therapeutic care pathways [4, 5]. At the regulatory level, several bills have been introduced in Parliament to recognize fibromyalgia as a chronic disabling disease, in line with recommendations from the European League Against Rheumatism [6] and the National Institute for Health and Care Excellence [7]. In addition to these international recommendations, a recent Italian consensus statement by the Neurological Society’s Special Interest Group on neuropathic pain has provided updated guidance on the management of fibromyalgia [8].

The estimated prevalence of fibromyalgia in the adult population ranges from 0.6% to 9.3%, depending on the diagnostic criteria applied [9, 10]. In Italy, available data indicate an average prevalence of approximately 6%, with a marked predominance in females.

The diagnostic process currently relies on criteria established by the American College of Rheumatology [11], which have evolved from 1990 to 2016 and combine clinical assessment with standardized self-report tools [12, 13], such as the Widespread Pain Index (WPI) and the Symptom Severity Scale (SSS). Despite advances in understanding fibromyalgia pathophysiology, including evidence from neuroimaging studies (e.g., fMRI and PET) and peripheral nervous system involvement such as small fiber pathology in a subset of patients, the lack of objective biomarkers means that diagnosis remains largely dependent on clinical expertise and professional training [14].

From a therapeutic standpoint, international guidelines agree on the need for a multidisciplinary and personalized approach, combining pharmacological treatments (e.g., duloxetine, pregabalin, amitriptyline) with non-pharmacological interventions [15, 16], (e.g., tailored physical exercise, cognitive-behavioral therapy, therapeutic education). However, the systematic adoption of such approaches is hindered by organizational disparities among regions, limited availability of specialized centers, absence of structured care networks, and gaps in professional training for healthcare personnel.

The high prevalence of fibromyalgia, the chronicity of symptoms, the negative impact on quality of life and work productivity, and the extensive use of healthcare and social services make this syndrome a significant public health issue. National and international studies indicate that FM patients undergo more medical visits, diagnostic tests, and treatments than the general population, resulting in considerable direct and indirect costs. This is further compounded by the psychological and economic burden on families and society as a whole [11].

In this context, the RF-2019–12369647 research project was launched with the strategic goal of standardizing and optimizing the clinical management of fibromyalgia nationwide. The study was designed around three main operational milestones:

i. nationwide mapping of Italian clinical centers involved in the care of patients with fibromyalgia;
ii. an exploratory review of existing national and international guidelines and care pathways;
iii. the development of shared educational and organizational resources to support harmonization of clinical practice. These objectives aim to bridge the gap between scientific evidence and clinical practice, thereby improving the effectiveness, equity, and sustainability of care pathways for patients with fibromyalgia.

## Materials and methods

The RF-2019–12369647 research project was designed as a multidimensional and integrated study aimed at describing the organizational landscape of fibromyalgia (FM) care in Italy. The project was structured into three main operational milestones:

(M1) nationwide mapping of public or publicly accredited clinical centers involved in FM care;
(M2) exploratory review of international and national diagnostic and therapeutic guidelines to support a comparative synthesis of recommendations;
(M3) development of a digital repository for the collection and dissemination of clinical, organizational, and educational resources.

Each milestone was addressed through a dedicated methodological approach, as detailed in the following subsections.

### Identification and mapping of clinical centers (M1)

A nationwide systematic survey was conducted to identify public or publicly accredited clinical centers involved in the diagnosis and management of fibromyalgia within the Italian National Health Service. Facilities operating exclusively in the private sector were excluded.

For the purposes of this study, “publicly accredited clinical centers” were defined as healthcare facilities formally authorized and reimbursed by the Italian National Health Service, regardless of whether fibromyalgia-specific outpatient clinics were formally designated.

The identification process was carried out in two phases.

- Phase 1: Web-based reconnaissance.
  An exploratory review of available online documentation (institutional websites, regional health authority publications, clinical networks, and publicly accessible hospital service listings) was conducted to compile a preliminary list of centers potentially involved in the care of patients with fibromyalgia.
- Phase 2: Institutional validation.
  The preliminary list was submitted to the Health Departments of all Italian Regions and Autonomous Provinces for verification and integration. This consultation resulted in an updated and expanded dataset, which, as of July 20, 2023, included 131 clinical centers distributed across the national territory.

### Nationwide survey (M1)

A structured, custom-developed questionnaire was distributed to the 131 identified clinical centers to collect quantitative and qualitative information on the organizational characteristics of fibromyalgia (FM) care. The questionnaire was developed by the project team and pilot-tested internally for clarity and relevance prior to dissemination.

The survey explored the following domains:

- organizational structure of the center (medical and allied health professionals involved, available specialties);
- care models adopted (presence of formally defined care pathways [PDTA], use of national or international guidelines, regional coordination);
- care workload (average number of patients managed annually, number of visits per patient);
- available diagnostic and therapeutic services;
- perceived organizational and clinical challenges in the management of patients with FM.

The questionnaire was administered electronically using the REDCap platform. Up to seven reminder emails were sent to non-responding centers to maximize participation. Of the 131 centers contacted, 56 completed the survey, corresponding to a response rate of 42.7%.

Collected data were analyzed using descriptive statistics (means, standard deviations, and percentage distributions) to identify prevailing organizational models, care practices, and critical issues across different regional contexts. Given the exploratory and descriptive nature of the survey, no inferential statistical analyses were performed.

### Review of scientific evidence and guidelines (M2)

An exploratory literature review was conducted to identify and summarize the main international and national diagnostic and therapeutic recommendations for fibromyalgia (FM), with the aim of supporting a comparative synthesis of existing guidance and informing subsequent project activities.

The review was performed by querying PubMed and Google Scholar using predefined search strings, including:

“diagnosis” AND “fibromyalgia”;

“treatment*” AND “fibromyalgia”;

“guideline*” AND “fibromyalgia”;

“differential diagnosis” AND “fibromyalgia”;

“nonpharmacological” AND “fibromyalgia”.

Peer-reviewed articles published in English, Italian, or French and indexed in the Science Citation Index were considered. Publications not relevant to the diagnostic or therapeutic focus of FM or lacking explicit methodological rigor were excluded. In addition, official guidelines and consensus documents produced by national and international institutions, including the European League Against Rheumatism, the National Institute for Health and Care Excellence, and the Italian Neurological Society [6–8, 11], as well as regional Italian care pathway documents identified through publicly accessible sources, were included.

Given the exploratory nature of the review, it was not preregistered and did not aim to provide a systematic appraisal or meta-analysis of the available evidence. Rather, the selected documents were organized into a structured bibliographic collection and used to produce a narrative summary and a comparative table of recommendations addressing diagnosis, treatment, and follow-up of FM.

### Digital repository (M3)

As part of the project activities, a cloud-based digital repository was developed as an operational support tool and shared among all project partners. The repository was designed to facilitate the collection, organization, and dissemination of clinical and organizational resources related to fibromyalgia care.

The repository includes:

- selected scientific literature on diagnosis, treatment, biomarkers, and follow-up;
- national and international guidelines;
- regional and local diagnostic–therapeutic care pathways (PDTA);
- a validated list of Italian clinical centers involved in FM care;
- a summary document synthesizing the main scientific evidence identified through the exploratory review.

In parallel, a dedicated web portal is under development to provide structured and regularly updated access to these resources. The platform is intended to offer differentiated access levels for healthcare professionals and patients, supporting education, information sharing, and clinical decision-making.

## Results

### Mapping of clinical centers for fibromyalgia (M1)

The mapping activity identified 131 public or publicly accredited clinical centers involved in the diagnosis and management of fibromyalgia, distributed across all 20 Italian Regions.

This result was achieved through a two-step process:

- Preliminary online reconnaissance, based on the analysis of publicly available sources, including institutional websites of Local Health Authorities (ASLs), hospitals, Scientific Institutes for Research, Hospitalization and Healthcare (IRCCS), and regional healthcare registries, to identify centers potentially involved in fibromyalgia care;
- Institutional validation, whereby the preliminary list was submitted to the Health Departments of the Italian Regions and Autonomous Provinces for confirmation, correction, or integration of the collected information.

As of July 20, 2023, seven Regions (Aosta Valley, Veneto, Tuscany, Marche, Abruzzo, Basilicata, and Lombardy) formally responded, providing official validation or updates to the identified centers.

Although not all Regions completed the institutional validation process, the combined web-based and institutional approach allowed for the identification of a nationwide network of centers involved in FM care, forming the basis for subsequent survey and analyses.

### National survey of centers: participation and organization (M1)

Of the 131 questionnaires distributed to the identified clinical centers, 56 were completed, corresponding to a response rate of 42.7%. The analysis of survey responses provided insights into regional organization, network integration, and staffing composition of centers involved in fibromyalgia (FM) care.

#### Regional distribution and care networks

Most responding centers (82%) reported the presence of other facilities managing patients with FM within the same Region, suggesting a relatively wide territorial coverage. However, only 16% of centers reported being part of a formally established regional care network, indicating limited structural integration among facilities. Furthermore, only 8% of centers identified the presence of a designated regional coordinating center, highlighting a lack of centralized leadership and coordination at the regional level.

#### Composition of healthcare personnel

Medical staff: Responding centers reported a mean of 6.27 physicians per center (range: 1–26). Rheumatologists were the most frequently represented specialty, present in 91% of centers, followed by internists (18%), neurologists (14%), and psychiatrists (9%).

Allied health professionals: Centers reported a mean of 7.3 allied health professionals per center (range: 0–45). Nursing staff were present in 89% of centers, while psychologists (21%) and physiotherapists (16%) were less frequently available.

The main organizational and clinical characteristics of the responding centers are summarized in Table 1.

**Table 1:** Organizational and clinical characteristics of responding centers involved in fibromyalgia care

Characteristic	Value
Number of responding centers	56
Mean number of physicians per center (range)	6.27 (1–26)
Rheumatologists, % of centers	91%
Neurologists, % of centers	14%
Internists, % of centers	18%
Psychiatrists, % of centers	9%
Mean number of allied health professionals per center (range)	7.3 (0–45)
Nurses, % of centers	89%
Psychologists, % of centers	21%
Physiotherapists, % of centers	16%
Mean number of patients per center (range)	485.3 (1–4,000)
Mean number of visits per patient/year (range)	2.36 (1–5)
Centers with internal access to diagnostic and therapeutic services	91%

Data are presented as percentages or means with ranges, as appropriate

### Care load and clinical activity

Responding centers reported a substantial care workload related to the management of patients with fibromyalgia.

The mean number of patients managed per center was 485.3, with a wide range spanning from 1 to 4,000 patients, reflecting marked heterogeneity in clinical activity across facilities.

The average number of visits per patient per year was 2.36 (range: 1–5), indicating regular follow-up for most patients with FM.

Regarding service availability, 91% of centers reported internal access to diagnostic procedures and therapeutic interventions, suggesting a generally adequate capacity for integrated clinical management within individual facilities.

### Adoption of guidelines and structured care pathways

A total of 21% of responding centers reported not adopting any formally defined diagnostic–therapeutic care pathway or written protocol for the management of fibromyalgia. This finding refers to the absence of structured and officially adopted care pathways (PDTA) or shared written protocols, rather than to the lack of use of established diagnostic criteria in routine clinical practice.

Among centers reporting the adoption of formal reference documents, the following distribution was observed:

- local or institutional care pathways (PDTA.A): 8.9%;
- regional care pathways (PDTA.R): 5.4%;
- national guidelines (LG.N): 12.5%;
- international guidelines (LG.I): 7.1%.

Only 8.9% of centers reported the concurrent adoption of structured care pathways together with both national and international guidelines, indicating substantial heterogeneity and limited harmonization in the implementation of diagnostic–therapeutic standards across centers.

The distribution of adopted care pathways and guideline-based reference documents among responding centers is detailed in Table 2.

**Table 2:** Adoption of diagnostic–therapeutic care pathways and guidelines among responding centers

Type of reference document	Centers (*n*)	Percentage (%)
No formally adopted protocol or care pathway	12	21.4
Local/institutional care pathway (PDTA.A)	5	8.9
Regional care pathway (PDTA.R)	3	5.4
National guidelines	7	12.5
International guidelines	4	7.1
Combination of care pathways and multiple guidelines	5	8.9

The absence of formal protocols refers to the lack of officially adopted written care pathways and does not imply the non-use of established diagnostic criteria in clinical practice

### Critical issues reported by centers

Nearly all responding centers (98%) reported at least one critical issue in the management of patients with fibromyalgia. The most frequently reported challenges are summarized below.

Organizational and coordination issues were the most commonly reported. Inadequate territorial coordination was cited by 69.6% of centers, reflecting limited integration among facilities and services at the regional level.

Training-related issues were also prominent. Low awareness of fibromyalgia among healthcare professionals and lack of structured training opportunities were both reported by 55.4% of centers, underscoring the need for targeted educational initiatives.

Limited availability and dissemination of updated guidelines was reported by 37.5% of centers, indicating difficulties in accessing shared and evidence-based reference documents.

Patient-related factors included low disease awareness among patients (30.3%) and difficulties in identifying official and reliable information channels (28.6%), highlighting the need for improved patient education and communication strategies.

Additional challenges, emerging from open-ended survey responses, included insufficient availability of standardized therapeutic protocols with high clinical effectiveness; poor adherence to non-pharmacological interventions; limited availability of dedicated medical and allied health professionals trained in fibromyalgia care; inadequate multidisciplinary integration, particularly among rheumatologists, neurologists, pain specialists, and mental health professionals; absence of structured rehabilitation pathways; and difficulties in delivering integrated care due to constraints in time, staffing, and organizational coordination.

Overall, these findings highlight the lack of a structured and homogeneous national network for fibromyalgia care and underscore the need for systemic and organizational interventions.

The main critical issues reported by responding centers are summarized in Table 3.

**Table 3:** Critical issues reported by responding centers in the management of fibromyalgia

Critical issue	Centers (*n*)	Percentage (%)
Inadequate territorial coordination	39	69.6
Low awareness of fibromyalgia among healthcare professionals	31	55.4
Lack of training opportunities for professionals	31	55.4
Limited availability of up-to-date guidelines	21	37.5
Low disease awareness among patients	17	30.3
Difficulty identifying official information channels	16	28.6

Centers could report more than one critical issue; therefore, percentages do not sum to 100%

### Review of scientific evidence and summary report (M2)

The following studies and guidelines were identified through a systematic literature review:

- 15 articles related to diagnostic approaches.
- 7 articles on potential biomarkers.
- 31 articles on pharmacological and non-pharmacological treatments.
- 2 articles concerning patient follow-up.

In addition, the following documents were collected:

- 12 international guidelines (e.g [1] [2] [3]).,
- 1 national guideline.
- 3 regional care pathways (PDTA) currently in use in Italy.

The data collected were synthesized into a technical report and a comparative synoptic table that contrasts diagnostic and therapeutic approaches at both national and international levels. This synthesis serves as the foundation for the future development of shared national operational recommendations.

### Development of the digital repository (M3)

As part of the third project milestone (M3), a cloud-based digital repository was developed to collect, organize, and disseminate clinical, organizational, and educational resources related to fibromyalgia care. The repository includes selected scientific literature, national and international guidelines, regional diagnostic–therapeutic care pathways (PDTA), a validated list of Italian clinical centers, and a summary document synthesizing the main scientific evidence identified through the exploratory review. The repository represents an operational tool intended to support knowledge sharing and harmonization of clinical practices.

## Discussion

The discussion is organized in accordance with the three main project milestones, integrating findings from the mapping and survey activities (M1), the exploratory review of guidelines (M2), and the development of shared organizational tools (M3). The findings obtained during the first 18 months of the RF-2019–12369647 project provide a comprehensive overview of the current organization of fibromyalgia (FM) care in Italy. The combined results of the nationwide mapping and the survey highlight a markedly heterogeneous landscape, characterized by organizational variability, inconsistent adoption of formal care pathways, limited coordination among services, and significant training gaps, all of which may negatively affect the continuity and effectiveness of care.

### Heterogeneity of care pathways and limited standardization

One of the most relevant findings is the limited and uneven adoption of structured diagnostic–therapeutic care pathways and formal guideline-based references. Although this does not imply the absence of established diagnostic criteria in daily clinical practice, the lack of formally adopted and shared protocols reflects the absence of a unified organizational framework for FM management. The coexistence of local, regional, national, and international reference documents, often applied in isolation, may contribute to variability in care delivery and territorial inequalities.

#### Fragmentation of territorial networks

The low proportion of centers integrated into formal regional networks and the limited presence of designated coordinating centers indicate a widespread lack of structured governance for FM care. Given the clinical complexity of FM and the need for multidisciplinary management, the absence of coordinated networks may increase the risk of fragmented care, duplication of interventions, and delays in diagnosis and treatment.

#### Training needs and professional challenges

Training-related issues emerged as a recurrent concern, with more than half of centers reporting insufficient awareness of FM among healthcare professionals and a lack of structured educational opportunities. This finding is particularly relevant in a clinical context where FM diagnosis and management rely heavily on clinical expertise and interdisciplinary collaboration. Limited training may hinder the appropriate integration of psychosocial and non-pharmacological components of care, which are strongly recommended by international guidelines.

#### Care burden and resource constraints

The reported clinical workload, with an average of over 480 patients per center and more than two visits per patient per year, underscores the substantial care burden associated with FM. In many centers, this workload does not appear to be supported by proportionate human resources, particularly with regard to allied health professionals. This imbalance may limit the ability to provide continuous, individualized, and multidisciplinary follow-up, especially for non-pharmacological interventions such as physiotherapy, psychological support, and therapeutic education.

#### Organizational implications and role of disciplines

An additional organizational aspect emerging from the survey is the predominance of rheumatology-based services among centers managing FM. While this reflects the historical clinical positioning of FM within rheumatology in Italy, it also highlights a potential misalignment with the growing recognition of FM as a disorder involving central and peripheral nervous system dysfunction. Strengthening collaboration between rheumatology, neurology, pain medicine, and mental health services may therefore represent a key step toward more integrated and pathophysiology-oriented care models.

#### Role of the repository and digital tools

The development of a shared digital repository and the ongoing implementation of a dedicated web portal represent operational tools aimed at improving access to updated clinical and organizational resources. Their potential impact will depend on sustained updating, integration within structured training programs, and effective dissemination among healthcare professionals, patients, and caregivers.

## Limitations

This study has several limitations. Not all Italian Regions completed the institutional validation of identified centers, and fewer than half of the mapped centers responded to the survey, which may limit the representativeness of the findings. In addition, the exploratory nature of the literature review did not allow for a formal systematic appraisal of evidence. These limitations should be considered when interpreting the results.

## Implications for health policy

Despite these limitations, the findings provide relevant insights into the organizational challenges of FM care in Italy. The absence of FM from the Essential Levels of Care (LEA), combined with regional variability and limited coordination, may contribute to inequities in access and quality of care. Addressing these issues will require coordinated action at national and regional levels, aimed at promoting shared care pathways, multidisciplinary integration, and continuous professional training.

## Conclusions

Through nationwide mapping, organizational assessment, and an exploratory review of scientific evidence, the RF-2019–12369647 project provides an updated overview of the organization of fibromyalgia care in Italy. The findings from the first 18 months highlight a fragmented clinical landscape, characterized by interregional variability, organizational gaps, and limited adoption of shared diagnostic–therapeutic standards.

The identification of 131 clinical centers involved in fibromyalgia management represents a relevant contribution to defining the operational context of care delivery, while also revealing inconsistencies in service organization, limited network integration, and a scarcity of coordinating structures. Survey data further indicate that many professionals operate in settings without formally adopted care pathways and face challenges related to training, disease awareness, and resource availability.

The exploratory review of scientific evidence supported the development of a structured synthesis of diagnostic and therapeutic recommendations. However, translating this body of knowledge into routine clinical practice remains dependent on organizational alignment, professional training, and institutional support.

Overall, these findings suggest the need for coordinated strategies aimed at promoting shared care pathways, strengthening multidisciplinary collaboration, enhancing continuous professional education, and improving access to reliable information for both healthcare professionals and patients.

Future phases of the project will focus on testing integrated care models and defining indicators to evaluate their clinical, organizational, and economic impact. In this perspective, the project may contribute to ongoing discussions on the recognition and organization of fibromyalgia care within the Italian National Health Service.
